# Ligature of the Formural Artery, for Popliteal Aneurism

**Published:** 1852-03

**Authors:** Daniel Brainard

**Affiliations:** Prof. of Surgery in Rush Medical College, etc.; Chicago


ARTICLE VIII.

*Ligature of the Formeral Artery, for Popliteal Aneurism—Recovery.* By DANIEL BRAINARD, M. D., Prof. of Surgery in Rush Medical College, etc.

A negro sailor, aet. 25 years, has noticed for about three years a pain in the ankle and back part of the leg, but only noticed a swelling in the ham about ten months ago. Such were the only facts I could gather on account of the obstuseness of his perceptions.

Jan. 12, 1852. The aneurism of the right limb occupied the popliteal space and formed a mass behind as large as the knee in front. Cold and compression have been attempted but abandoned on account of the indocility of the patient, who having no idea of the danger would submit to little restraint.

Operation performed in presence of the Class at the College, the ligature being applied about the middle of the thigh upon the fermural artery.

Nothing unusual occurred after or during the operation. No coldness of the foot followed and no pulsation returned in the tumor.

Jan. 23d. An abscess which had formed above the wound in the thigh opened into the wound.

Feb. 13th. Ligature came out.

Feb. 23d. Wound healed; swelling subsided. Tumor firm, about half its size before the operation, and diminishing.

*Chicago, Feb.*, 1852.

